# Crosstalk of Alzheimer’s disease-phenotype, HPA axis, splenic oxidative stress and frailty in late-stages of dementia, with special concerns on the effects of social isolation: A translational neuroscience approach

**DOI:** 10.3389/fnagi.2022.969381

**Published:** 2022-09-15

**Authors:** Aida Muntsant, Lydia Giménez-Llort

**Affiliations:** ^1^Institut de Neurociències, Universitat Autònoma de Barcelona, Barcelona, Spain; ^2^Department of Psychiatry and Forensic Medicine, School of Medicine, Universitat Autònoma de Barcelona, Barcelona, Spain

**Keywords:** Alzheimer’s disease, neuroimmunoendocrine crosstalk, aging, 3xTg-AD mice, cognition, neuropsychiatric symptoms, translational neuroscience, social isolation

## Abstract

Coping with emotional stressors strongly impacts older people due to their age-related impaired neuroendocrine and immune systems. Elevated cortisol levels seem to be associated with an increased risk of cognitive decline and dementia. In Alzheimer’s disease (AD), alterations in the innate immune system result in crosstalk between immune mediators and neuronal and endocrine functions. Besides, neuropsychiatric symptoms such as depression, anxiety, or agitation are observed in most patients. Here, we studied the psychophysiological response to intrinsic (AD-phenotype) and extrinsic (anxiogenic tests) stress factors and their relation to liver, kidneys, heart, and spleen oxidative status in 18-months-old female gold-standard C57BL/6 mice and 3xTg-AD mice model for AD. The emotional, cognitive, and motor phenotypes were assessed under three different anxiogenic conditions. Survival, frailty index, and immunoendocrine status (corticosterone levels and oxidative stress of peripheral organs) were evaluated. Genotype differences in neuropsychiatric-like profiles and cognitive disfunction in 3xTg-AD females that survived beyond advanced stages of the disease persisted despite losing other behavioral and hypothalamic–pituitary–adrenal (HPA) physiological differences. A secondary analysis studied the impact of social isolation, naturally occurring in 3xTg-AD mice due to the death of cage mates. One month of isolation modified hyperactivity and neophobia patterns and disrupt the obsessive-compulsive disorder-like digging ethogram. Frailty index correlated with spleen organometrics in all groups, whereas two AD-specific salient functional correlations were identified: (1) Levels of corticosterone with worse performance in the T-maze, (2) and with a lower splenic GPx antioxidant enzymatic activity, which may suppose a potent risk of morbidity and mortality in AD.

## Introduction

Alzheimer’s disease (AD) is a complex and multi-factorial disorder caused by the interaction of biological and environmental factors, where age-related changes play a determinant role (Llewellyn et al., 2019; Knopman et al., 2021). The complex aging process leads to old age being the most heterogeneous period of life. In AD, the complexity of patients’ clinical profiles (Kaufer et al., 1998; Kamiya et al., 2014) and the heterogeneity in the temporal progression of the disease highlight the relevance of personalized-based interventions, especially in the advanced stages of the disease (Komarova and Thalhauser, 2011).

Dementia is associated with increased mortality (van Dijk et al., 1991) and although it is not yet well-understood, experimental, clinical, and epidemiological evidence suggest that crosstalk interactions between the brain and the peripheral or systemic abnormalities might have a crucial role in the development and progression of AD (Wang et al., 2017). According to the AD neuroimmunomodulation hypothesis, alterations in the innate immune system result in a dysregulation of the crosstalk between immune mediators and neuronal function both at central and peripheral levels (Giménez-Llort et al., 2008, 2012; Maccioni et al., 2009). Coping with emotional stressors has also a stronger impact on older people due to their age-related impaired neuroendocrine and immune systems (Kiecolt-Glaser et al., 2002). In the last years, the relevance of social relationships’ effects on morbidity and mortality outcomes has been reported, taking particular importance during the COVID-19 pandemic (Gerst-Emerson and Jayawardhana, 2015; Donovan and Blazer, 2020). Social isolation and loneliness are particularly relevant among older adults due to functional declines, health morbidities, or relationship losses (Steptoe et al., 2013; Gerst-Emerson and Jayawardhana, 2015). Regarding AD, social isolation not only increases the risk of developing dementia (Kuiper et al., 2015; Read et al., 2020), but it is also associated with an increased memory decline, negatively affecting cognitive performance, and worsening neuropsychiatric symptoms (NPS) (Friedler et al., 2015; Porcelli et al., 2019; Curelaru et al., 2021).

Experimental gerontologists highlight the relevance of using aged animals to mimic the complexity and multi-factorial aging processes in humans (Turner et al., 2012; Mitchell et al., 2015), so that the translational approach provides immediate clues to fast human neuroscience research. Among the animal models of AD, we have proposed long-term female survivors of the widely used 3xTg-AD mice as a model for mortality selection bias and heterogeneity in end-of-life dementia (Torres-Lista et al., 2017). Based on the familial AD mutations PS1/M146V and APPSwe, and harboring tauP301L human transgene, this model progressively develops temporal- and regional-specific neuropathological patterns observed in the human brain of AD patients (Oddo et al., 2003; Belfiore et al., 2019). Cognitive deficits (Billings et al., 2005; Sterniczuk et al., 2010), a broad spectrum of NPS-like symptoms (Giménez-Llort et al., 2007; España et al., 2010), and impairment in neuro-immunoendocrine status that correlate with worse NPS-like behavioral profiles (Giménez-Llort et al., 2008; Gimenez-Llort et al., 2014) have been described.

Since the establishment of 3xTg-AD and non-transgenic (NTg) mouse colonies in our laboratory, we have repeatedly observed that the animals overcoming 15 months of age show milder impairment of cognitive and NPS-like behaviors than expected for their neuropathological status. Sex differences in mortality rates (Giménez-Llort et al., 2008; Arranz et al., 2011; Blázquez et al., 2014), made females survivors a singular scenario (Torres-Lista et al., 2017). The present work further explores previously observed behavioral and functional phenotype convergence in long-term 3xTg-AD female survivors (Torres-Lista et al., 2017). Here, we studied the cognitive, motor and NPS-like phenotypes of 18-month-old females assessed under different anxiogenic conditions. This age corresponds to old age in NTg mice and late stages of the disease in 3xTg-AD mice. Survival and indicators of frailty phenotype and neuro-immunoendocrine status (as determined by corticosterone levels, oxidative stress of peripheral organs, and organometrics) were also measured. In a secondary analysis, we studied the impact of short-term social isolation in a subgroup of age-matched female 3xTg-AD mice.

## Materials and methods

### Animals

A total of twenty-six 18-month-old (18.7 ± 0.16) female mice from the Spanish colonies of homozygous 3xTg-AD (*n* = 17) and NTg (*n* = 9) mice on a C57BL/6J background (after embryonic transfer and backcrossing at least 10 generations) established in the Universitat Autònoma de Barcelona (Baeta-Corral and Giménez-Llort, 2014) were used in this study. The animals were maintained under standard laboratory conditions of food and water ad lib, 22 ± 2°C, 12-h light/dark cycle with lights on at 8:00 a.m., and 50–60% relative humidity.

### Social conditions

Animals of the same genotype were maintained in groups of 3–4 mice per cage (Macrolon, 35 cm × 35 cm × 25 cm). Since at these advanced stages of the disease, increased mortality rates in 3xTg-AD mice occur (Giménez-Llort et al., 2008), in a secondary analysis, a set of 3xTg-AD mice living in social isolation (*n* = 9, one animal living alone in one cage for 1 month) was also studied. When isolated, animals still were socially connected through olfaction and audition, and as in the group-housed conditions, the cages were enriched with nesting materials. The study complies with the ARRIVE guidelines developed by the NC3Rs (Kilkenny et al., 2010).

### Behavioral assessment

A behavioral battery of tests evaluates locomotion/exploration, anxiety-like behaviors, emotionality, and cognitive functions under three different anxiogenic conditions: anxiogenic direct exposure to an illuminated field with/without objects, mild neophobia in a new home cage, and black corridors of a maze resembling burrows (Giménez-Llort et al., 2007). Behavioral assessments were performed blind to the experiment, in a counterbalanced manner, in the light cycle, from 09:00 to 13:00 h.

Day 1: Corner Test (CT) and Open Field Test (OF)—The animal was placed in the center of a clean standard home cage, and neophobia was evaluated for 30 s through the number of corners visited, rearings, and latency to perform the first rearing. Immediately after the CT, mice were placed in the center of an open field (beige metal drawer, 42 cm × 38 cm × 15 cm) and observed for 5 min. The sequence of behavioral events that define the animal’s ethogram was recorded as follows: duration of freezing (latency to move), latency to leave the central square and enter the peripheral ring, latency, and total duration of self-grooming behavior. Horizontal (distance covered) and vertical (rearings) locomotor activities were recorded for each minute of the test. Distance, time in the center/periphery, and immobile time were evaluated by video-tracking analysis (ANY-Maze 5.14). Defecation boli and urination were also recorded.

Day 2: Context and Object Recognition Test (OF2OR)- The day after, the animals were re-tested in the open field (OF2) to evaluate their response when confronting the same anxiogenic environment again. Analysis of activity was done during the first minute of the test, the period where neophobia is more manifested, and we have previously described AD-genotype differences (Torres-Lista et al., 2014). Immediately after, animals were moved to a standard home cage, where they remained for 1 min before being reintroduced in the OF (a known environment) and were assessed for their ability to recognize a familiar object (S, sample) from a new one (N) with the novel object recognition test (OR) (Giménez-Llort et al., 2021). In the sample trial, the animals were let explore two identical objects, S1 and S2 (glass bottles, 15 cm × 12 cm, 5 cm diameter), equally spaced until they reached the 20 s exploration criteria until a maximum time of 600 s. Two hours thirty minutes later, animals were reintroduced to the apparatus for 5 min (test trial), where two different non-explored objects were located: an identical copy of the sample objects (S3) and a completely new object (N, rectangular aluminum can, 15 cm × 10 cm × 4 cm). Preference for the new object was measured.

Day 3: Marble test (MB)—Mice were placed individually for a 30-min period in a standard home cage containing nine glass marbles (1 cm × 1 cm × 1 cm) evenly spaced, making a square. The number of marbles intact (untouched), moved or half-buried, and buried (completely hidden) were counted as previously described (Torres-Lista et al., 2015).

Day 4: T-maze (TM)—Working memory was assessed in a black T-shaped maze (woodwork; two short arms of 30 cm × 10 cm and a long arm of 50 cm × 10 cm). One forced-choice (acquisition trial) was followed by one free-choice (recall trial) with a 60 s intertrial interval. Latencies of turn, reach the intersection, crossed the intersection (four paws criteria), and the time elapsed until the mice completed the 20 s in the forced arm (time to reach the criteria) were recorded. The animals that completed the trial in less than the cut-off time (10 min) were allowed to explore the maze (both arms accessible) in the recall trial for 5 min. The choice of the already visited arm in the previous trial was considered an error, and the total number was calculated. The time spent reaching the correct arm, defecation boli, and urination were also recorded.

### Survival, body weight, and mouse clinical frailty index

Survival was recorded continuously with a daily cadence. After the behavioral assessment, the body weight was recorded to monitor the physical status of the animals. Frailty was assessed using an adaptation of the Mouse Clinical Frailty Index (Whitehead et al., 2014), including 30 “clinically” assessed non-invasive items.

### Immunoendocrine status and oxidative stress of liver, kidneys, heart, and spleen

Blood samples and organs tissue were collected during the euthanasia. Serum was obtained by centrifugation and stored at −80°C. Immunoendocrine status was monitored through spleen weight and index (relative weight, % of body weight). Corticosterone content (ng/mL) was analyzed using a commercial kit (Corticosterone EIA kit from Immunodiagnostic Systems) and read at 450 nm of absorbance with Varioskan LUX ESW 1.00.38. The antioxidant capacity was studied from liver, kidneys, heart, and spleen homogenates. Total glutathione (GSH) levels were assayed by the enzymatic recycling method by monitoring the change in absorbance at 412 nm adapted to 96-well plates. Glutathione reductase (GR) enzymatic activity was assessed following the oxidation of NADPH spectrophotometrically at 340 nm for 300 s. For the GPx activity, the reaction was followed spectrophotometrically by the decrease of the absorbance at 340 nm for 300 s (Muntsant and Giménez-Llort, 2021).

### Statistics

Statistical analyses were performed using SPSS 15.0 software. Data are presented as mean ± SEM or percentage. Differences between two different genotypes were evaluated with Student’s *t*-test while Chi-square or Fisher’s exact test for incidences. Subgroups comparisons were conducted with ANOVA followed by Bonferoni *post-hoc* test. In temporal courses paired *t*-tests were used for within-subject analysis. Pearson’s correlation analyzed the pathological/behavioral correlates. The survival curve was analyzed with the Kaplan-Meier test. In all the tests, *p* < 0.05 was considered statistically significant.

## Results

**FIGURE 1:**
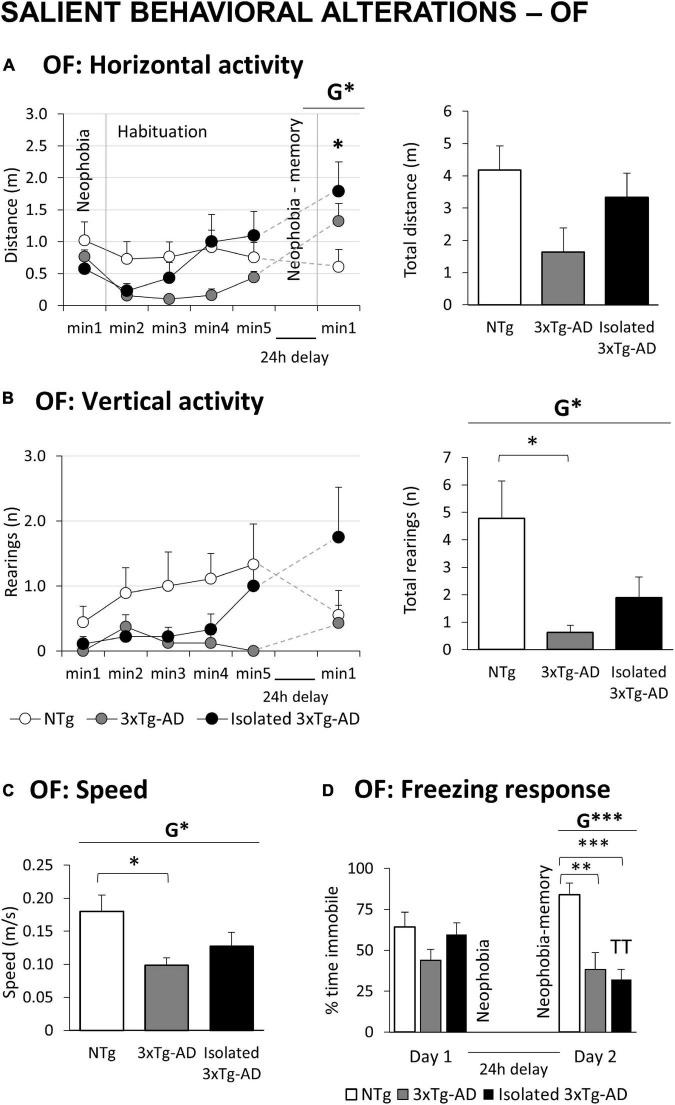
Salient behavioral alterations in 18-month-old female 3xTg-AD mice in the open field tests compared to NTg females with normal aging. Results are expressed as mean ± SEM. **(A)** OF: Open field test Horizontal activity: left, time course; and right, total distance; **(B)** vertical activity: left, time course; and right, total number; **(C)** speed; **(D)** freezing response. Statistics: Students *t*-test: G, genotype effect, G *p < 0.05, G **p < 0.01, G ***p < 0.001. ANOVA for comparisons between all the groups of mice followed by Bonferroni’s *post-hoc* test: *p < 0.05 and ***p < 0.01 vs. the NTg-group. Paired *t*-test, TT *p* < 0.01 vs. the same group on Day 1.

**FIGURE 2:**
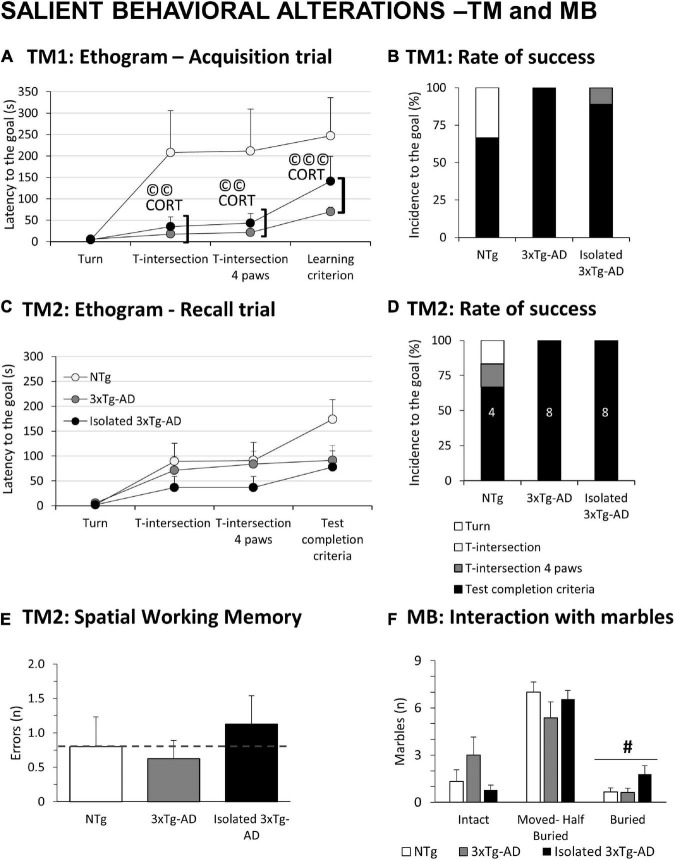
Salient behavioral alterations in 18-month-old female 3xTg-AD females in the T-maze and marble test compared to NTg females with normal aging. Results are expressed as mean ± SEM or incidence (%). **(A)** Ethogram in the acquisition trial (TM1) in the T-Maze; **(B)** rate of success in TM1; **(C)** ethogram in the recall trial (TM2) in the T-Maze; **(D)** rate of success in TM2; **(E)** spatial Working memory in TM2; **(F)** MB: Marble test, Interaction with marbles. Inset of bars, number of animals exhibiting the behavior. Statistics: Students *t*-test: ^#^, isolation effect, ^#^*p* < 0.05. Pearson’s correlation analysis, ©©*p* < 0.01; ©©*p* < 0.001; CORT: corticosterone levels.

**FIGURE 3:**
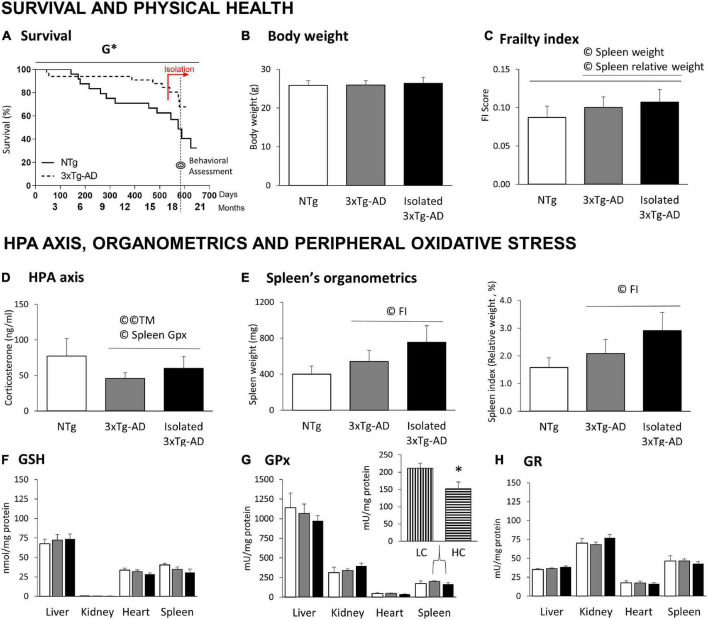
Survival, physical health, HPA axis, and peripheral oxidative stress in 18-month-old 3xTg-AD females compared to NTg females with normal aging. **(A)** Survival; **(B)** body weight; **(C)** frailty index scores; **(D)** HPA axis measured by Corticosterone levels; **(E)** organometrics of spleen: weight and spleen index (relative weight, % vs. body weight); Oxidative Stress of liver, kidney, heart, and spleen: **(F)** GSH levels; **(G)** GPx enzymatic activity and **(H)** GR enzymatic activity. Results are expressed as the mean ± SEM. HC (high corticosterone levels), LC (low corticosterone levels). Statistics: Kaplan-Meier and Log-Rank test; G, genotype effect, G *p < 0.05. Student *t*-test, *p < 0.05 vs. LC counterparts. Pearson’s correlation analysis, ©*p* < 0.05; ©©*p* < 0.01; TM, T-maze test; FI, frailty index.

The results, depicted in Figures 1–3, and Table 1, show the psychophysiological response to intrinsic and extrinsic stress factors, hypothalamic–pituitary–adrenal (HPA) axis, and peripheral status (organometrics and oxidative stress).

**TABLE 1:** Convergence of 18-month-old NTg and 3xTg-AD females’ phenotypes in the corner test, open field test and object recognition test.

	NTg mice (*n* = 9)	3xTg-AD (*n* = 8)		3xTg-AD-ISO (*n* = 9)		
	Mean ± SEM	Mean ± SEM	G	Mean ± SEM	G	ISO
**Corner test (30 s)**						
Visited corners (*n*)	7.22 ± 0.88	5.88 ± 1.27	*n.s*	5.56 ± 0.85	*n.s*	*n.s*
Rearing (latency, s)	17.89 ± 3.02	16.63 ± 3.81	*n.s*	13.11 ± 3.32	*n.s*	*n.s*
Rearings (number)	1.44 ± 0.29	1.88 ± 0.58	*n.s*	2.56 ± 0.41	*n.s*	*n.s*
**Open field test (Day 1, 5 min)**						
Initial freezing (latency, s)	20.56 ± 13.79	3.00 ± 0.63	*n.s*	5.00 ± 0.87	*n.s*	*n.s*
Exit of the center (latency, s)	28.33 ± 13.98	9.75 ± 1.94	*n.s*	44.78 ± 31.96	*n.s*	*n.s*
Entrance to the periphery (latency, s)	78.33 ± 37.12	64.75 ± 31.60	*n.s*	94.33 ± 41.35	*n.s*	*n.s*
Vertical activity (latency, s)	161.78 ± 38.45	175.38 ± 41.39	*n.s*	210.22 ± 33.49	*n.s*	*n.s*
Bizarre, vertical rearing (latency, s)	265.33 ± 26.1	207.88 ± 42.5	*n.s*	295.89 ± 4.1	*n.s*	*n.s*
Self-grooming (latency, s)	161.00 ± 26.49	147.50 ± 27.36	*n.s*	161.33 ± 28.44	*n.s*	*n.s*
Self-grooming (*n*)	1.33 ± 0.29	1.25 ± 0.25	*n.s*	1.00 ± 0.24	*n.s*	*n.s*
Defecation boli (*n*)	2.78 ± 0.46	3.63 ± 0.84	*n.s*	3.00 ± 0.50	*n.s*	*n.s*
**Context and object recognition test Open field test (Day 2, 1 min)**						
Initial freezing (latency, s)	20.11 ± 8.12	9.71 ± 2.53	*n.s*	7.00 ± 2.15	*n.s*	*n.s*
Exit of the center (latency, s)	23.44 ± 7.80	17.29 ± 3.99	*n.s*	13.38 ± 4.39	*n.s*	*n.s*
Entrance to the periphery (latency, s)	23.44 ± 8.52	24.57 ± 6.22	*n.s*	23.50 ± 7.77	*n.s*	*n.s*
Vertical activity (latency, s)	50.67 ± 6.72	53.86 ± 4.09	*n.s*	40.50 ± 7.62	*n.s*	*n.s*
Bizarre, vertical rearing (latency, s)	60.00 ± 0.00	60.00 ± 0.00	*n.s*	60.00 ± 0.00	*n.s*	*n.s*
Self-grooming (latency, s)	56.67 ± 3.33	60.00 ± 0.00	*n.s*	59.00 ± 0.94	*n.s*	*n.s*
Self-grooming (*n*)	0.33 ± 0.17	0.00 ± 0.00	*n.s*	0.14 ± 0.13	*n.s*	*n.s*
Defecation boli (*n*)	1.22 ± 0.46	0.29 ± 0.17	*n.s*	0.14 ± 0.13	*n.s*	*n.s*
**Object recognition test (Day 2, 10 min)**						
Latency 1st object (s)	142.67 ± 76.52	60.29 ± 42.74	*n.s*	60.13 ± 19.48	*n.s*	*n.s*
Latency 2nd object (s)	235.00 ± 65.45	362.57 ± 85.02	*n.s*	238.00 ± 80.77	*n.s*	*n.s*
Time 1, acquisition criteria (s)	551.00 ± 40.01	490.71 ± 50.53	*n.s*	408.88 ± 73.64	*n.s*	*n.s*
Total touch duration (s)	10.50 ± 2.58	12.14 ± 2.95	*n.s*	14.12 ± 2.35	*n.s*	*n.s*
Animals to reach acquisition criteria	1/9	3/8	*n.s*	4/9	*n.s*	*n.s*

Statistics: Results are expressed as mean ± SEM or incidence. Student t-test, G, genotype effect (vs. NTg mice), ISO, isolation effect in 3xTg-AD mice (vs. 3xTg-AD mice housed in groups). All n.s., p > 0.05.

### Salient behavioral alterations despite phenotypic convergence

Figures 1A,B illustrates the temporal curves (left) and total accumulated counts (right) of horizontal and vertical exploratory activity during the 5 min in the OF and the first min in the repeated test. A decrease in vertical exploration was observed in 3xTg-AD mice compared to NTg mice (Figure 1B, G*, *p* = 0.036). In the horizontal component, the sensitive variable was the walking speed, with 3xTg-AD groups walking slower than NTg mice (Figure 1C, G*, *p* = 0.016). In the repeated test, a burst of initial locomotor performance was observed in 3xTg-AD animals, reaching statistical significance in the horizontal component (Figure 1A, G*, *p* = 0.031). The immediate freezing response (neophobia) during the first seconds of the test was analyzed (Figure 1D). No differences were observed on the first day. However, compared to NTg mice, this second test recorded a significant decrease in 3xTg-AD mice (G^***^, *p* = 0.000).

The subgroup of isolated 3xTg-AD did not differ from their counterparts housed in groups (Table 1 and Figure 1). Still, a slight increase in activity and speed (Figures 1A–C) counteracted the low activity phenotype characteristic of 3xTg-AD mice, reducing the changes to discriminate alterations compared to NTg animals. Their freezing response in the re-test (Figure 1D) was statistically significant as compared to their previous neophobia response in the open-field test (*^TT^p* = 0.002) and differed from that of NTg mice with higher significance than did group-housed 3xTg-AD mice (^***^p = 0.000).

As detailed in Table 1, all the other variables measured in the CT and the OF test showed striking similarities between genotypes. Concerning the OR, most animals were unsuccessful in reaching the acquisition criteria (rate of success: NTg: 1/9; 3xTg-AD: 3/8; isolated 3xTg-AD: 4/9). However, the ethogram concerning the latencies to touch the objects for the first time, and Time 1 (acquisition criteria, 600 s for those failing to do so) can be reported. In all the groups, a high individual variability that resulted in a high statistical variance in most variables was noticeable.

Figure 2A depicts the ethograms performed in the two trials of the TM. As detailed in Figure 2B, only 6/9 NTg mice achieved the learning criteria, while the coping-with-stress strategy of the other three was to rest with their back protected at the starting point. In contrast, all the 3xTg-AD mice (8/8) completed the first free trial. The proactive strategy of isolated 3xTg-AD mice resulted in only 2/9 mice being unable to complete the test. In the recall test (Figure 2C), animals that completed the first trial were evaluated. Again, all 3xTg-AD mice completed the test, whereas 4/6 NTg mice achieved the test completion criteria (1/6 did not even start) (Figure 2D). The number of spatial working memory errors (revisiting an explored area) was recorded in those animals able to perform the task. Albeit the sample size was not enough to reach statistical significance, an increased number of errors in spatial alternation was noted in the isolated 3xTg-AD mice (Figure 2E). Statistically significant Pearson’s correlations, specific of 3xTg-AD mice in their ethogram in the acquisition trial with corticosterone levels (©CORT) were found (T-intersection, *r* = 0.691, *p* = 0.002; T-intersection four paws, *r* = 0.695, *p* = 0.002; Learning criterion, *r* = 0.761, *p* = 0.000). In the MB, isolation broke the habitual digging ethogram with an increased number of buried marbles compared to the other groups (Figure 2F, ^#^*p* = 0.047).

### Survival, physical health, and frailty index

Figure 3A illustrates the survival curves of an initial sample of 57, 24 NTg, and 33 3xTg-AD mice from birth to euthanasia. Log-rank analyses showed genotype differences [G*, χ^2^(1) = 4.319, *p* = 0.038]. In this cohort, the survival drops for NTg females started at adulthood (5 months) and decreased continuously until the end of the experiment (19 months), with a survival rate of 32.47%. In contrast, 3xTg-AD females exhibited an early 10% mortality rate before young adulthood (2 months), but their survival rate at the end of the experiment was 67.76%. Two isolated 3xTg-AD females died during the isolation period compared to one group-housed. The day before euthanasia, two NTg females died. Four NTg and seven 3xTg-AD mice were used for other experiments.

No differences were found in the body weight (Figure 3B) and the frailty index scores (Figure 3C) despite a slight increase in the index in 3xTg-AD females and its enhancement in those with isolation. As detailed below, frailty index correlated with the splenic organometrics (weight and relative weight) in all cases (©spleen weight: all mice, *r* = 0.416; only 3xTg-AD mice, *r* = 0.545; ©spleen index, all mice, *r* = 0.407; only 3xTg-AD mice, *r* = 0.452; *p* < 0.05 in all cases).

### Convergence but Alzheimer’s disease-dependent crosstalk between HPA axis, behavior, and peripheral organs

No genotype differences were found in corticosterone levels (Figure 3D), but a slight (*n.s*.) increase in isolated 3xTg-AD females. In the 3xTg-AD mice, corticosterone levels were correlated to the ethogram in the TM (Figure 2A, as detailed before) and the splenic GPx activity (Figure 3G, *r* = −0.547, *p* = 0.028).

The spleen size (weight) and index (relative size, % vs. body weight) were recorded as an indirect measure of their physiological/healthy status. No differences were found, but a slight (*n.s*.) increase of splenomegaly incidence in the AD genotype (NTg: 2/9, 3xTg-AD: 5/8, isolated 3xTg-AD: 6/9). Statistically significant Pearson’s correlations indicated that both variables correlated with the frailty score (Figure 3C,(© FI, as detailed before).

No differences between the experimental groups were found in the antioxidant parameters of four different organs (Figures 3F–H). However, we performed a secondary segregation analysis since a correlation between corticosterone levels and spleen GPx was found in 3xTg-AD mice. Animals with corticosterone levels above the mean (HC, high corticosterone, *n* = 9) showed a significant decrease in splenic GPx activity compared to their low corticosterone (LC, below the mean, *n* = 8) counterparts (*p = 0.016).

## Discussion

Exclusion or under-representation of older individuals is not unusual in clinical trials despite being the most significant health care resource (Zulman et al., 2011). At the preclinical level, most experimental designs are performed in young adulthood, some in middle age. The reduced sample size of survivors, with the concomitant increase in laboratory costs and the complex heterogeneity of age-related scenarios, produce a scarcity of research in older animals, even more in models of neurodegenerative disease (Fahlström et al., 2011; Shoji et al., 2016; Jackson et al., 2017; Torres-Lista et al., 2017).

Our laboratory described for the first time the convergence of behaviors of 3xTg-AD and NTg mice as a part of the complex old age-related scenarios and the heterogeneity regarding physical, cognitive, and anxiety-like patterns (Torres-Lista et al., 2017). In the present work, the convergence was confirmed in almost all variables analyzed, and we also showed that the poor performance of the aged NTg animals also contributed to it. Despite the convergence, the 2-days open field paradigm and the MB test allowed to detect salient behavioral alterations in the 3xTg-AD mice and/or the effects of isolation. Also, AD-specific salient functional correlations were identified among the enhanced anxious-like profile and HPA axis and the worse splenic oxidative stress, pointing at AD-derangement in this brain-peripheral psychophysiological triad as a potent risk of morbidity and mortality in AD.

Cognitive alterations were shown under anxiogenic conditions when animals were re-tested in the anxiogenic OF, agreeing with previous reports in 14-month-old animals (Muntsant et al., 2021). In this 2-days open field paradigm (Torres-Lista et al., 2014), the ethogram and time course of the first experience allows to measure a reduced activity in the 3xTg-AD. In contrast, in the first minute of the second session shows an altered response in the 3xTg-AD mice characterized by a need to explore. This is agreement with results in scenarios mimicking end-of-life dementia (Muntsant et al., 2021). In the case of isolated 3xTg-AD females, there was an hyperactive effect in agreement with recently described increase of fine and gross motor function in male sex (Muntsant and Giménez-Llort, 2020), and non-purposed behaviors mimicking obsessive-compulsive disorder (OCD) (Gimenez-Llort and Alveal-Mellado, 2021). High activity with low freezing response was present in 3xTg-AD females and enhanced in the isolated subgroup. Working memory deficits were enhanced in isolated females, albeit they did not reach statistical significance. Similar results were recently reported on isolated males (Muntsant and Giménez-Llort, 2020). However, their increased burying was noticeable and disrupted the ethogram signature, an effect we recently reported in isolated 3xTg-AD males (Gimenez-Llort and Alveal-Mellado, 2021) with translational value regarding the impact of isolation OCD. Thus, like in humans (Gerst-Emerson and Jayawardhana, 2015), housing/social conditions can significantly impact animal behavior, morbidity, and mortality outcomes. However, only a few laboratories have studied the effects of poor social housing in AD models, despite reporting worsened cognition and neuropathology (Pietropaolo et al., 2009; Huang et al., 2015; Muntsant and Giménez-Llort, 2020; Peterman et al., 2020), enhancement of olfactory patterns (Marín-Pardo and Giménez-Llort, 2021), body and organs weight loss, and increased asymmetric atrophy of the hippocampus (Muntsant and Giménez-Llort, 2020). Interestingly, the present work shows that even short isolation could increase the activity in 3xTg-AD mice and counteract the low activity levels associated with the AD-genotype. This also warns of potential confounding factors and false negatives when experimental groups of mutant animals include isolated animals, a common practice due to the high incidence of this housing condition due to several reasons (i.e., aggressivity and mortality).

We have consistently reported a sex-dependent increase in the vulnerability of 3xTg-AD mice (Giménez-Llort et al., 2010; Torres-Lista et al., 2017) and a survival bias (Giménez-Llort et al., 2021). Therefore, due to the higher variability of the aging process, the survival curves are a must to understand in which scenario the results are referred. More recently, we have reported cohorts that offered a distinct survival scenario (Muntsant et al., 2021; Castillo-Mariqueo and Giménez-Llort, 2022). In this case, the survival analysis showed genotype-dependent increased mortality rates in the NTg mice, but a survival bias in 3xTg-AD was associated with a severe initial survival drop before adulthood (2 months of age). In APP23 females, we recently reported also the relevance of survival bias to define the very-old age experimental scenarios (Giménez-Llort et al., 2021). As mentioned, the study of the age-matched NTg female with this survival scenario also provides data on a scarcely reported normal aging process in gold-standard C57BL/6J strain.

The frailty index is becoming a common tool to measure health status as it seems sensitive to predicting disease outcomes and mortality (Zeng et al., 2015). At the translational level, the Mouse Clinical Frailty Index (Whitehead et al., 2014; Feridooni et al., 2015) is also a valuable tool in longevity and aging studies in mice. In these 18-month-old cohorts, no differences were found, but a trend to increase in 3xTg-AD females and the isolated subgroup correlated with those in spleen organometrics. The higher frailty scores were observed in the integument and muscular-skeletal systems, such as alopecia, loss of whiskers, and dermatitis, which are the more common frailty clinical presentations in aged mice (Pettan-Brewer and Treuting, 2011; Muntsant and Giménez-Llort, 2021).

Mice isolation has been proposed as an animal model for self-induced body weight loss (van Leeuwen et al., 1997), being males significantly affected (Guo et al., 2004) as we also recently demonstrated in 3xTg-AD mice after a naturalistic long-term social isolation (Muntsant and Giménez-Llort, 2020). In the present work, the sexual dimorphism of body size and the shorter period of isolation could explain the lack of differences.

Crosstalk between endocrine abnormalities of the HPA axis and patients with AD has been repeatedly described (Ouanes and Popp, 2019). Interestingly, in these old 3xTg-AD females, the corticosterone levels correlated with worse performance in the TM and a poor splenic GPx capacity, which would agree with the HPA axis as one of the major pathways involved in stress response (Nelson, 1972) and changes in the glucocorticoid’s levels favoring alterations in the immune response (Costa-Pinto and Palermo-Neto, 2010). Despite the convergence in the HPA axis and oxidative stress profiles, HC 3xTg-AD females showed lower splenic GPx antioxidant activity than LC counterparts. This data would also agree with findings suggesting that the effects of stressful events on the immune system depend on the individual anxiety response produced by the situation (Cruces et al., 2014).

Regarding the convergence, it would agree with our first report on 15-month-old animals, where a significant increase in plasma corticosterone was observed only in males (Giménez-Llort et al., 2008) associated with their higher vulnerability/mortality ratios. The possible existence of protective or regulatory mechanisms in 3xTg-AD female survivors could also explain the convergence with controls observed in the present work. A trend of increased corticosterone levels in the isolated subgroup of 3xTg-AD females was observed but did not reach statistical significance, probably due to the short isolation period. In fact, the duration of the isolation is an important influence on the neuroendocrine response (Cacioppo et al., 2015). Concerning oxidative stress status, we have described early peripheral alterations in male and female 3xTg-AD mice, with a decrease in antioxidant defenses in the spleen, kidney, and liver (de la Fuente et al., 2012). In the present work, a decrease in antioxidants associated with the normal aging process, as described in 15- and 18-months-old animals (Martínez De Toda et al., 2020) could also explain the convergence in the oxidative stress profiles found here when comparing old NTg females with 3xTg-AD survivors.

Spleen and liver dysfunction reflected as splenomegaly (Giménez-Llort et al., 2008; Marchese et al., 2014; Yang et al., 2015) and hepatomegaly (Fraile-Ramos and Giménez-Llort, 2021) respectively, have been reported in this animal model. We proposed organometry as an early indicator of peripheral immunological system aging and their easy weight measurement by echography as a clinical translation (Giménez-Llort et al., 2012; Gimenez-Llort et al., 2014). In the present work, the size and index of the spleen and liver [as an immunological organ (Racanelli and Rehermann, 2006)] were recorded as an indirect measure of animals’ immunological/physiological status. Interestingly, in a longitudinal study in 3xTg-AD females, from 2 to 15 months of age, we reported function and redox state of peritoneal leukocytes as preclinical and prodromic markers, but convergence of many immune-oxidative stress markers with those of NTg females at the oldest studied age (Maté et al., 2015). Thus, the results indicated changes in CD25 + B and naïve CD8 T cells percentage, NK percentage and cytotoxic activity, GSSG/GSH ratio, and GSH content already at asymptomatic stages (2 months of age). Premature immunosenescence at 4 months of age, when intraneuronal β-amyloid immunoreactivity emerges, with reduced chemotaxis, phagocytosis, and lymphoproliferation and also lower reduced glutathione and higher xanthine oxidase activity. Alterations in lymphoproliferation, phagocytosis, xanthine oxidase activity and CD5+ B1 cells continued at 15 months of age. However, CD11b+, CD335+, CD19+, CD25+, macrophage and lymphocyte chemotaxis, basal lymphoproliferation, natural killer activity, GSH, GSSG/GSH ratio and catalase activity of 3xTg-AD mice and NTg mice converged at this old age.

Despite the organometrics and oxidative status of spleen in 3xTg-AD females did not reach statistical significance, their correlation with frailty score and HPA axis suggests that even a short isolation in 3xTg-AD female survivors may increase their risk of morbidity and mortality. In fact, various meta-analyses have described that social isolation is associated with an increased risk of cardiovascular disease and stroke (Holt-Lunstad and Smith, 2016; Valtorta et al., 2016), and it is considered a risk factor for all-cause mortality (Holt-Lunstad et al., 2015).

At translational level, in accordance with the data presented, several systemic abnormalities have been also described, reflecting underlying disease processes (Wang et al., 2017). Identifying and better understanding this crosstalk might offer new chances for develop diagnostic biomarkers or new treatments against AD.

## Conclusion

The scarcity of research in older animals caused by the complex age-related scenario and the high individual heterogeneity mirrors the clinical research scenarios, and it is a strong limitation to addressing critical gerontological/geriatric issues. Further phenotypical and behavioral studies in normal aging and AD animal models may help to understand better this complex scenario and identify vulnerability/resilience factors. The main findings of this work refer to cognitive, physical, and anxiety-like profiles in 3xTg-AD females that survived beyond advanced stages of the disease but were hardly distinguishable from normal aging counterparts in almost all variables analyzed. Despite this convergence, AD-salient behaviors and crosstalk with the HPA axis and spleen support the relevance of studying brain-periphery interactions. On the other hand, in a subgroup of 3xTg-AD females, short-term isolation modified anxiety patterns and cope with stress strategies. The correlations and segregation analysis according to corticosterone levels revealed a negative impact on the antioxidant GPx enzymatic activity in the spleen, which may suppose a potential risk of morbidity and mortality. Further studies should investigate the relationship between the immunoendocrine status, and the underlying neuroanatomical substrates related to AD, especially in emotion-related brain regions, such as amygdala or ventral hippocampus.

## Data availability statement

The raw data supporting the conclusions of this article will be made available by the authors, without undue reservation.

## Ethics statement

The study was conducted according to the guidelines of the Declaration of Helsinki and approved by the Ethics Committee of Departament de Medi Ambient i Habitatge, Generalitat de Catalunya (CEEAH 3588/DMAH 9452) on March 8, 2019. The study complies with the ARRIVE guidelines developed by the NC3Rs and the aim to reduce the number of animals used.

## Author contributions

LG-L: conceptualization, funding acquisition, and writing—review and editing. AM: performance and analysis of behavior, statistical analysis, illustrations, and writing—original draft, review, and editing. Both authors have read and agreed to the published version of the manuscript.
